# Antipsychotics lower peripheral markers of inflammation in drug-naïve early psychosis: a pilot study

**DOI:** 10.3389/fpsyt.2026.1769162

**Published:** 2026-02-17

**Authors:** Nicole Šafářová, Marián Kolenič, Ivana Tašková, Václav Čapek, Petra Fürstová, Filip Španiel

**Affiliations:** 1 Early Episodes of SMI Research Center, National Institute of Mental Health, Klecany, Czechia; 2 Department of Clinical Pharmacy, 3rd Faculty of Medicine, Charles University, Prague, Czechia; 3 Psychiatric Hospital Bohnice, Prague, Czechia; 4 Faculty of Pharmacy, Charles University, Hradec Králové, Czechia

**Keywords:** antipsychotic medication, cumulative chlorpromazine equivalents, early psychosis, first-episode schizophrenia, monocyte-to-lymphocyte ratio, neuroinflammation, neutrophil-to-lymphocyte ratio, platelet-to-lymphocyte ratio

## Abstract

**Introduction:**

Neuroinflammation is increasingly recognized as a core pathophysiological mechanism in schizophrenia and can be indirectly assessed through peripheral inflammatory markers. Therefore, this pilot study investigated the impact of antipsychotic treatment on inflammation in patients with first-episode psychosis (FEP) who were antipsychotic-naive at study entry.

**Methods:**

Thirty-three drug-naïve FEP patients provided blood samples upon admission (V0) and follow-up (V1), from which peripheral inflammatory markers—i.e., neutrophil/lymphocyte ratio (NLR), monocyte/lymphocyte ratio (MLR), platelet/lymphocyte ratio (PLR), and systemic immune-inflammation index (SII)—were calculated. Antipsychotic doses during continuous hospitalization between V0 and V1 (34 days, IQR: 21–49 days) were converted into cumulative chlorpromazine equivalents (cCPZ).

**Results:**

In multiple regression models adjusting for sex, age, BMI, DUP, and clozapine use, cumulative antipsychotic exposure significantly predicted reductions in ΔNLR (p = 0.048), ΔMLR (p = 0.041), ΔPLR (p = 0.028), and ΔSII (p = 0.028). All associations remained significant following false discovery rate adjustment (pFDR = 0.048 for all outcomes).

**Conclusion:**

These findings suggest a consistent dose-dependent anti-inflammatory effect during early antipsychotic treatment in FEP. Given the exploratory nature of this study, larger studies are needed to confirm these findings.

## 1 Introduction

Schizophrenia is a severe mental illness affecting ~1% of the global population (1) with significant functional impairment and substantial disability (2, 3). Traditionally, schizophrenia has been conceptualized within the dopamine hypothesis, which attributes positive symptoms to the hypodopaminergic activity in subcortical regions (4). This theory guides current treatment centered on dopamine D2 receptor antagonism (5), which is effective for positive symptoms but largely ineffective in managing negative symptoms and cognitive decline (6)—key determinants of functional outcomes. As a result, current treatment alleviates only a fraction of the total disease burden (7, 8), highlighting the need to investigate additional pathophysiological mechanisms beyond classical neurotransmission.

In this context, the immune dysregulation and neuroinflammatory pathways offer a compelling framework for understanding the pathophysiological changes in schizophrenia that may lead to the symptom progression and greater disease severity (9–13). Moreover, the evidence of immune involvement has been observed from the earliest stages of psychotic illnesses (14)—individuals at clinical high risk (CHR) and with first-episode psychosis (FEP) show increased inflammatory markers, including IL-6 and NLR (10, 15–19)—and could also be linked to the neurodegenerative changes in schizophrenia (20, 21).

Mechanistically, cellular stress can activate innate immune pathways, leading to the release of cytokines and chemokines via signaling cascades such as TLR and NF-κB (22–24). These signals can recruit and activate peripheral immune cells, thereby linking central immune processes to systemic immune alterations (23). Consistent with this model, elevated peripheral indices such as NLR, MLR, PLR, and SII have been repeatedly reported in FEP (16, 17, 25).

At the same time, antipsychotic medications (APs) appear to have effects that extend beyond neurotransmission and may represent a unique opportunity to influence immune processes relevant to schizophrenia. Growing evidence suggests that APs may modulate inflammatory pathways, including cytokine and chemokine signaling and microglial activity, potentially through intracellular immune-related mechanisms such as NF-κB and TLR pathways (26, 27). Meta-analyses demonstrate that second-generation antipsychotics reduce levels of pro-inflammatory cytokines in patients with FEP, including IL-6, IL-1β, TNF-α, IFN-γ, and IL-2 (13, 18, 28).

These findings suggest that antipsychotics may exert clinically relevant immunomodulatory effects within the central nervous system, where immune processes are thought to contribute to illness persistence and progression (26, 27). Consistent with this, preclinical studies indicate that modulation of immune pathways by antipsychotics may influence behavioral domains relevant to negative and cognitive symptoms (29–31), although direct clinical evidence in humans remains limited.

Despite this potential, the effects of APs on routinely available peripheral inflammatory markers such as NLR, MLR, PLR, and SII remain inconsistent and insufficiently understood (13, 16, 32), limiting their current translational utility. Given these uncertainties, a more precise understanding of whether commonly available inflammatory markers reflect antipsychotic-related immunomodulation is needed. This question is particularly relevant in drug-naïve patients with FEP, where immune alterations can be studied before the confounding effects of chronic illness and long-term antipsychotic exposure.

Therefore, the present study aimed to evaluate whether cumulative AP treatment is associated with changes in NLR, MLR, PLR, and SII in drug-naïve FEP patients. To our knowledge, this is the first study to explore the anti-inflammatory action of APs in FEP using cumulative chlorpromazine dose equivalents (cCPZ) as an assessment of drug exposure across diverse regimens while controlling for confounders such as age, sex, clozapine use, and duration of untreated psychosis (DUP).

## 2 Methods

### 2.1 Study setting

This retrospective pilot study analyzed data from the ongoing ESO project at the National Institute of Mental Health, Klecany, Czech Republic.

Initial drug-naive blood samples (V0) were collected at the Psychiatric Hospital Bohnice in Prague and represent the first blood sample obtained upon each patient’s admission to inpatient hospitalization. The follow-up assessment (V1) was conducted at the National Institute of Mental Health in Klecany after clinical stabilization, which resulted in variability in the V0-V1 interval across patients.

Data for this study were extracted from the ESO database in January 2023. All participants provided written informed consent, and the study was conducted in accordance with the latest version of the Declaration of Helsinki. The study protocol was reviewed and approved by the Research Ethics Board of the National Institute of Mental Health in Klecany (Approval No. 127/17).

### 2.2 Inclusion criteria

- Drug-naïve patients with the first-episode psychosis (FEP).
- Continuous hospitalization between V0 and V1.
- Medication and daily doses had to be consistently monitored throughout their hospitalization.
- No ongoing viral or bacterial infections between V0 and V1.

These criteria led to the inclusion of 33 drug-naïve FEP patients in this study.

### 2.3 Data collection

Continuous hospitalization allowed for consistent monitoring of medication adherence. During the study period, administration of antipsychotic medication alongside dosage information was recorded daily. All doses were converted into chlorpromazine equivalents, and cumulative doses were calculated for each patient to facilitate comparison across all of the study participants.

Blood samples were collected at two predefined time points—V0 and V1. Standard complete blood counts (CBCs) were provided, including the absolute count of neutrophils, lymphocytes, monocytes, and platelets. Hemato-immunological markers, including NLR, MLR, PLR, and SII, were computed using the following formulas:

- NLR = neutrophils/lymphocytes.
- MLR = monocytes/lymphocytes.
- PLR = platelets/lymphocytes.
- SII = (platelets × neutrophils)/lymphocytes.

For each marker, the change between the two time points (Δ = V1 − V0) was calculated and used as the dependent variable in the subsequent analysis. The SII was included because it reflects a combined index of neutrophil, lymphocyte, and platelet counts, offering a broaderand potentially more sensitive measure of systemic inflammation relevant to psychotic disorders. Higher values of NLR, MLR, PLR, and SII are generally interpreted as indicators of elevated peripheral inflammation. Therefore, a decrease in these markers is suggestive of a reduction in inflammatory activity.

### 2.4 Covariate selection

Cumulative antipsychotic exposure (cCPZ) was the prespecified primary predictor of interest. All other variables were included to account for their potential confounding effects on inflammation. Specifically, sex was included due to the known sex differences in immune response, with males typically exhibiting higher basal inflammation (33, 34). Age was included given its established association with increased inflammation (35, 36). Although the evidence for duration of untreated psychosis (DUP) is inconclusive, it was included due to its possible association with inflammation in early psychosis (37–39). Clozapine use was included to account for the potential confounding effect of clozapine-induced neutropenia (CIN) and its other known alterations of white blood cells. Finally, body mass index (BMI) was included to control for the metabolic contribution to low-grade inflammation (40).

### 2.5 Data analysis

Associations between cumulative antipsychotic exposure and changes in inflammatory markers (ΔNLR, ΔMLR, ΔPLR, ΔSII) were examined using multiple regression models including sex, age, BMI, duration of untreated psychosis (DUP), and clozapine use as covariates. Regression assumptions were evaluated visually using residual diagnostics. False discovery rate (FDR) adjustment (Benjamini–Hochberg) was applied across the four outcomes to account for multiple testing. Bonferroni-adjusted *p*-values are reported in **Supplementary Table 1** for transparency but were not used to guide inference due to correlated endpoints and the exploratory nature of this pilot study.

All statistical analyses were performed using the R software (version 4.2.1). The dataset was screened for outliers and missing values; no patients were excluded, and no imputation was performed. Analyses included only complete cases. Statistical significance was set at *p* < 0.05 (two-tailed).

### 2.6 Power and sample size

This study was designed as an exploratory pilot to evaluate feasibility and estimate effect sizes for future research; therefore, no *a priori* power analysis was conducted. However, *post hoc* sensitivity and power analyses were done in the R software. With a total sample size of N = 33 and 6 predictors (cCPZ, sex, age, DUP, BMI, and clozapine use), this study had 80% power (α = 0.05) to detect large effects of approximately Cohen’s f² = 0.521 (R² = 0.343) for the predictor set. Therefore, smaller or moderate effects may have gone undetected due to the limited power.

## 3 Results

### 3.1 Cohort analysis

The analysis included 33 drug-naïve FEP patients, of whom 11 were female and 22 were male. The mean age was 24.58 ± 5.43 years. The median time between V0 and V1 was 34 days (IQR: 21–49 days). For more demographic information, see **Table 1**.

**Table 1:** Descriptive statistics for demographic and clinical variables (N = 33).

Variables	Mean ± SD	Median [IQR]
DUP (days)		27.00 [14.00, 116.00]
age at V0 (years)	24.58 ± 5.43	
Length of V0 to V1 (days)		34.00 [21.00, 49.00]
Length of AP treatment to V0 (days)	6.33 ± 12.75	
BMI	21.94 ± 2.85	

V0—the initial visit, V1—the follow-up visit, DUP—the duration of untreated psychosis, AP—antipsychotic.

Descriptive statistics for inflammatory markers at V0 and V1 are depicted in **Table 2**. The overall changes in NLR (mean Δ = 0.13, *p* = 0.576), MLR (mean Δ = 0.017, *p* = 0.395), and SII (mean Δ = 31.6, *p* = 0.605) were minimal and not statistically significant. Only PLR showed a significant increase (mean Δ = 24.4, *p* = 0.002), likely influenced by the high variability across patients (Δ range: –48.6 to +170.0).

**Table 2:** Descriptive statistics for inflammatory markers at baseline (V0) and follow-up (V1).

Marker	Mean V0 (SD)	Mean V1 (SD)	Mean Δ	*p*-value
NLR	1.89 (1.03)	2.02 (1.23)	0.13	0.576
MLR	0.228 (0.0831)	0.245 (0.114)	0.017	0.395
PLR	105 (31.1)	130 (41.8)	24.4	0.002**
SII	421 (282)	453 (293)	31.6	0.605

**statistical significance p < 0.01.

NLR—neutrophil-to-lymphocyte ratio, MLR—monocyte-to-lymphocyte ratio, PLR—platelet-to-lymphocyte ratio, SII—systemic immune-inflammation index, V0—the initial visit, V1—the follow-up visit.

**Figure 1:**
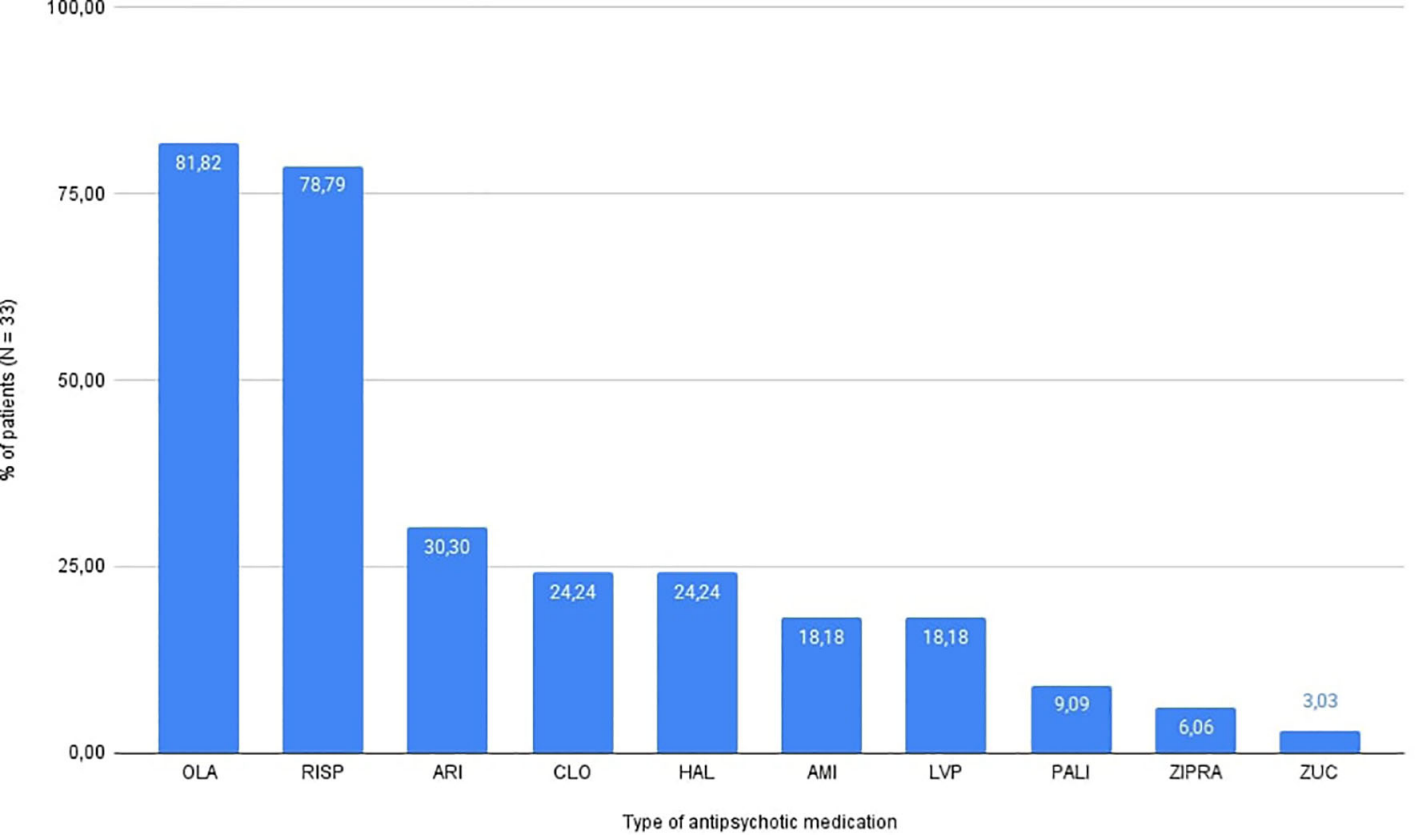
Distribution of antipsychotic use in patient cohort (N = 33). OLA, olanzapine; RISP, risperidone; ARI, aripiprazole; CLO, clozapine; HAL, haloperidol; AMI, amisulpride; LVP, levomepromazine; PALI, paliperidone; ZIPRA, ziprasidone; ZUC, zuclopenthixol.

The median cumulative antipsychotic exposure expressed in chlorpromazine equivalents (cCPZ) in the whole cohort was 16331 mg (IQR: 8114–46076 mg), while the mean cCPZ was 28370.1 mg. The majority of patients were prescribed olanzapine (81.8%, n = 27) and risperidone (78.8%, n = 26). Aripiprazole was used in 30.3% of patients (n = 10), clozapine and haloperidol both in 24.2% (n = 8), amisulpride and levomepromazine in 18.2% (n = 6), paliperidone in 9.1% (n = 3), ziprasidone in 6.1% (n = 2), and zuclopenthixol in 3.0% (n = 1). Throughout the entire study period (V0 to V1), 42.4% of patients (n = 14) were treated with antipsychotic monotherapy. The distribution of antipsychotic use is depicted in **Figure 1**, highlighting predominant use of olanzapine and risperidone among the patients, both of which are recommended as first-line treatments in psychiatric guidelines (41) for managing acute psychosis and schizophrenia.

### 3.2 Association between cumulative antipsychotic exposure and inflammation

In multiple regression models with Δ inflammatory markers as outcomes (Δmarker ~ cumulative CPZ-equivalents + sex + age + BMI + DUP + clozapine use), cumulative antipsychotic exposure significantly predicted reductions in ΔNLR (β = −2.22×10^-5^, 95% CI: −4.31×10^-5^ to −1.19×10^-6^, *p* = 0.048), ΔMLR (β = −1.71×10^-6^, 95% CI: −3.26×10^-6^ to −1.52×10^-7^, *p* = 0.041), ΔPLR (β = −7.83×10^-4^, 95% CI: −1.44×10^-^³ to −1.25×10^-4^, *p* = 0.028), and ΔSII (β = −6.47×10^-^³, 95% CI: −1.19×10^-^² to −1.04×10^-^³, *p* = 0.028). All associations remained statistically significant following FDR adjustment (*p*FDR = 0.048 for all outcomes), although close to the conventional significance threshold of *p* < 0.05. Full regression results are presented in **Table 3**. Furthermore, none of the associations remained statistically significant after Bonferroni correction (see **Supplementary Table 1**).

**Table 3:** Adjusted regression models predicting change in inflammatory markers.

Δ-marker	β	95% CI	*p*	*p*FDR
NLR	-2.22e-05	[-4.31e-05, -1.19e-06]	0.0484*	0.0484*
MLR	-1.71e-06	[-3.26e-06, -0.15e-06]	0.0409*	0.0484*
PLR	-7.83e-04	[-1.44e-03, -1.25e-04]	0.0277*	0.0484*
SII	-6.47e-03	[-1.19e-02, -1.04e-03]	0.0276*	0.0484*

*statistical significance p < 0.05.

Cumulative exposure: unscaled β and p-values. Covariates: sex, age, BMI, DUP, and clozapine use.

NLR, neutrophil-to-lymphocyte ratio; MLR, monocyte-to-lymphocyte ratio; PLR, platelet-to-lymphocyte ratio; SII—systemic immune-inflammation index.

To improve interpretability, regression coefficients were also expressed per +10,000 mg-days CPZ-equivalent exposure. On this scale, an additional +10,000 mg-days was associated with estimated reductions of −0.22 in ΔNLR, −0.02 in ΔMLR, −7.83 in ΔPLR, and −64.66 in ΔSII, indicating largest cCPZ–driven reductions in SII. Additionally, sex significantly predicted ΔMLR (*p* = 0.045; *p*FDR = 0.049), with males showing lower reductions compared to females. Age, BMI, DUP, and clozapine use were not statistically significant predictors for any inflammatory marker.

## 4 Discussion

This pilot study provides a unique perspective on the intricate relationship between antipsychotic therapy and inflammation in patients with FEP. By examining within-subject changes in inflammatory markers throughout their treatment, we extend previous research by moving beyond static, cross-sectional comparisons to a more dynamic assessment of immunological shifts during antipsychotic therapy. Our findings indicate that cumulative chlorpromazine equivalent doses were associated with reductions in peripheral inflammation markers (NLR, MLR, PLR, and SII), suggesting a potential dose-dependent anti-inflammatory effect of APs. Although the associations survived FDR correction, the effect estimates were imprecise, consistent with an exploratory pilot sample.

Our findings of dose-dependent reductions in NLR, MLR, PLR, and SII following antipsychotic treatment in initially drug-naive patients may reflect a reduction in peripheral immune activation, which could represent a downstream component in the pathophysiological cascade of psychosis. One plausible mechanism by which antipsychotics may modulate inflammatory pathways is the modulation of the TLR-4/NF-κB cascade, through which antipsychotics may suppress glial activation and cytokine release (26, 27, 42, 43). In parallel, their potential anti-inflammatory action may involve dopaminergic modulation—particularly via D3 receptor-mediated astrocyte activation (44–47) and subsequent NF-κB signaling. Although the potential anti-inflammatory effects of antipsychotics may vary, particularly between the first- and second-generation agents—with evidence suggesting stronger anti-inflammatory action for SGAs (13, 18, 48)—we did not conduct a comparative analysis due to limited statistical power. Nonetheless, the observed dose-dependent effect based on cumulative chlorpromazine-equivalent dosing supports, at present, a general anti-inflammatory role for antipsychotics as a class, warranting further investigation.

Despite the promising findings, our study has several limitations. The small sample size (N = 33) limits the statistical power and generalizability—our study was only powered to detect large effects (f² ≈ 0.52) with 80% power. As such, our findings should be interpreted with caution. The retrospective design introduces potential biases related to timing, unmeasured confounders, and variability in clinical or laboratory data. Although we adjusted for key covariates (age, sex, DUP, BMI, and clozapine use) and excluded patients with acute viral or bacterial infections, other factors—such as comorbidities or somatic medications—could not be fully controlled for. These limitations call for more rigorous prospective research on larger cohorts. A further limitation is the treatment heterogeneity; all antipsychotics were grouped into a single cCPZ variable, regardless of their classification as either first-generation (FGA) or second-generation (SGA) subgroups. This approach facilitated standardized dose comparisons; however, it likely masked potential class-specific immunomodulatory effects, as previously noted (SGAs vs. FGAs). Future studies should stratify analyses by antipsychotic class to examine these differences more accurately. Another limitation concerns the interrelated nature of the inflammatory markers examined. The SII is mathematically derived from platelet, neutrophil, and lymphocyte counts, and therefore partly reflects the same signal captured by NLR and PLR. This inherent overlap may inflate associations or limit the ability to interpret each marker as an independent indicator of immune activity. Nonetheless, we included SII because it has been increasingly recognized as an integrative marker of systemic inflammation, even though most psychosis research to date has focused more extensively on NLR and related ratios. Finally, although all results survived FDR correction, they did not remain significant under the more conservative Bonferroni adjustment, underscoring the exploratory nature of the findings. Due to these limitations, our findings should be viewed as an initial signal that requires confirmation rather than definitive evidence of antipsychotic-mediated immunomodulation.

An additional aspect that warrants consideration is the metabolic impact of second-generation antipsychotics. Many SGAs are associated with weight gain, dyslipidemia, and insulin resistance (49), which are themselves linked to chronic low-grade inflammation (50). Over longer treatment periods, these metabolic effects may counteract or obscure potential early anti-inflammatory effects of antipsychotic medications. Given the relatively short follow-up interval in the present study and the young, largely metabolically healthy cohort, the observed associations likely reflect early treatment-related immunomodulatory effects rather than longer-term metabolic consequences. Future longitudinal studies incorporating metabolic measures and longer follow-up periods will be necessary to disentangle the dynamic interplay between antipsychotic exposure, metabolic changes, and inflammation.

Clinically, inflammatory markers, such as the NLR, MLR, PLR, and SII, are appealing due to their low cost and broad availability, although their clinical utility has not yet been established. If validated in larger, prospective cohorts, these markers could serve as accessible and cost-effective tools for early detection, prognostication, and personalized intervention—particularly in identifying individuals at high risk for psychosis or poor treatment response. They could also help in detecting subclinical relapses and guide personalized antipsychotic strategies. Elevated baseline levels of these hemato-immunological markers might be indicative of a heightened risk for the development of psychosis. Therefore, future work may clarify whether early changes in these markers could inform intervention strategies. Importantly, our findings underscore the need to further explore the anti-inflammatory potential of APs. By focusing on their immunomodulatory properties, we may be able to develop novel approaches and strategies that could potentially treat the refractory symptoms of schizophrenia.

Thus, future research should focus on prospective, longitudinal studies to determine whether longitudinal changes in peripheral inflammatory markers during antipsychotic treatment have clinical relevance, for example in relation to symptom persistence, relapse, treatment response or alterations in brain structure.

## 5 Conclusion

This exploratory pilot study suggests that cumulative antipsychotic exposure may be associated with modest within-subject reductions in peripheral markers of inflammation, such as NLR, MLR, PLR, and SII, in patients with drug-naive first-episode psychosis.

Our results align with the emerging view that antipsychotics may have a dual role in psychosis treatment—beyond their neurotransmitter effects, they could modulate inflammation, offering new insights into the pathophysiology and management of schizophrenia. However, due to this study’s several key limitations, such as small sample size, retrospective design, and the borderline statistical significance after correction, its results necessitate cautious interpretation. Therefore, these results should be considered as preliminary and hypothesis-generating. Thus, larger, prospective longitudinal studies will be required to confirm whether dose-dependent anti-inflammatory effects of antipsychotics are robust, clinically meaningful, and relevant to treatment outcomes.

## Data Availability

The data analyzed in this study is subject to the following licenses/restrictions: The dataset contains sensitive clinical information from human participants and cannot be shared publicly due to privacy and ethical restrictions. Available on reasonable request. Requests to access these datasets should be directed to Nicole Šafářová, nicole.safarova@nudz.cz.
