# Supplementary material for: Antipsychotics lower peripheral markers of inflammation in drug-naïve early psychosis: a pilot study
Source: Front Psychiatry. 2026 Feb 17;17:1769162. doi: 10.3389/fpsyt.2026.1769162 (PMC12953376; doi:10.3389/fpsyt.2026.1769162)
Supplement: Supplementary file 1 [file Table1.docx]

*Bonferroni adjustment*

| **Marker** | **Raw p** | **Bonferroni p_adj** |
| --- | --- | --- |
| ΔNLR | 0.0484* | 0.1936 |
| ΔMLR | 0.0409* | 0.1636 |
| ΔPLR | 0.0277* | 0.1108 |
| ΔSII | 0.0276* | 0.1104 |

** statistical significance p>0.05*
